# Food quantity and quality shapes reproductive strategies of *Daphnia*

**DOI:** 10.1002/ece3.9163

**Published:** 2022-08-01

**Authors:** Anna Bednarska

**Affiliations:** ^1^ Department of Hydrobiology, Institute of Functional Biology and Ecology, Faculty of Biology University of Warsaw Warsaw Poland

**Keywords:** clonal differences, *daphnia*, food quantity, food quality, phenotypic plasticity, resource allocation

## Abstract

In freshwater environments, one of the challenges aquatic grazers face are periods of suboptimal food quantity and quality. In a life table experiment, the effects of food quantity (a gradient of algae concentration) and quality (a diet of cyanobacteria) on the life histories and resource allocation strategy in *Daphnia magna* were tested. Growth‐related traits were similarly affected under different food regimes while the reproductive strategies differed in animals exposed to low food quantity and quality. The per‐clutch investment (clutch volume) did not differ between *Daphnia* fed with cyanobacteria and underfed mothers, but resources were differently allocated; underfed mothers increased their per‐offspring investment by producing fewer, but larger eggs, whereas cyanobacteria‐fed mothers invested in a greater number of eggs of smaller size. I argue that both strategies of resource allocation (number vs. size of eggs) may be adaptive under the given food regime. The results of the study show that the cyanobacteria diet‐driven fitness losses are comparable to losses caused by food quantity, which is only slightly above the growth capability threshold for *Daphnia*.

## Graphical abstract

Cyanobacteria diet causes as severe fitness losses as does food abundance which is only slightly above the threshold for growth of Daphnia. Strategies of resources’ allocation in low food quality and quantity differs.

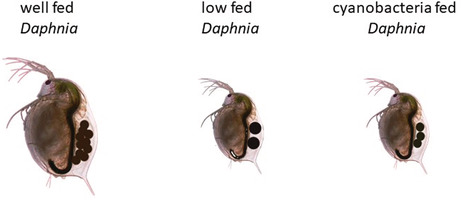

## 1 INTRODUCTION

Communities of planktonic primary producers in temperate lakes change in species composition and in size structure in a yearly cycle. The sequence of events in plankton succession is described by the PEG model (Sommer et al., 1986, 2012). In essence, the PEG model highlights two maxima of phytoplankton biomass separated by a clear water phase, where phytoplankton biomass is extremely low. The first maximum, the spring bloom, is typically dominated by small‐sized forms (mainly diatoms), whereas the summer bloom is usually dominated by large‐sized, and often toxic, filamentous, or spherical taxa such as cyanobacteria. As a result, the feeding conditions for planktonic herbivores change accordingly, that is, at the beginning of the season there is a period of abundance of high‐quality food (reach in essential compounds like sterols and fatty acids), followed by a period when food is extremely scarce, and later when the summer maximum of biomass starts to build up, the phytoplankton community is dominated by taxa that constitute suboptimal food for their consumers. Low‐quality food can be characterized by low caloric value, inadequate elemental composition, lack of essential compounds, poor manageability, and the presence of toxic compounds (e.g., Bednarska et al., 2014; Boersma & Kreutzer, 2002; Ger et al., 2014; von Elert et al., 2003). Thus, during one vegetation season, or even during the lifetime of a single generation, herbivorous planktonic animals face periods of changing food quality and quantity (from shortage to superfluous).

Among the planktonic herbivores, cladocerans of the genus *Daphnia* are among the most efficient grazers of phytoplankton. However, their grazing efficiency depends greatly upon phytoplankton abundance and taxonomic composition (Gliwicz, 1980). Hence, both food quantity and quality can be strong selective factors shaping grazer communities and can determine the efficiency of energy transfer between trophic levels.

The importance of food quantity and quality in shaping the fitness of *Daphnia* has been demonstrated in a number of studies. Boersma and Kreutzer (2002) found that the food quality in terms of phosphorus content (P‐limited vs. P‐rich algae) can determine the threshold food concentration for *Daphnia* growth. Sarpe et al. (2014) studied the effects of food quantity and stoichiometric quality on *Daphnia* fitness and found that the quantity of food is important only when high quality food is offered, and when low‐quality food was provided, even its high abundance could not compensate for its low quality. This indicates that the quality of food is as important in determining *Daphnia* fitness as the amount of available food. In another study on the interactions between food quantity and quality, it was shown that somatic growth of *D. magna* fed high food quantity of low cholesterol content (low quality) was not significantly different from growth of daphnids fed low food quantity of high cholesterol content (Lukas & Wacker, 2014a). The authors of the latter study also showed that *Daphnia* is able to perform the regulative response to low food quality by changing its carbon budget only when high amount of food is offered.

The quality of food can be characterized not only by its elemental composition, but also by the presence or lack of other essential compounds which cannot be synthesized de novo by crustaceans (e.g., sterols; Goad, 1981). As cyanobacterial cells contain no sterols and limited amounts of PUFAs (polyunsaturated fatty acids; e.g., von Elert et al., 2003), they have low nutritional value. Both, sterols and PUFAs are precursors of hormones and other bioactive compounds, and building the cell membranes, are crucial for cladocerans growth and reproduction (Martin‐Creuzburg & von Elert, 2009). Moreover, not only feeding on cyanobacteria, but even their mere presence in the environment can be growth‐limiting for *Daphnia*. The ability of cyanobacteria to synthesize toxins and filamentous or spherical morphology of their colonies (causing mechanical interference in the feeding process) adds to their low quality as food source for zooplankton (e.g., see review by Ger et al., 2014).

Low food quantity and poor food quality related to the presence of cyanobacteria can impose similar restraints on the fitness of *Daphnia*. It was shown that they both trigger changes such as delayed reproduction, decreased growth rate, and fecundity (Pires et al., 2011; Repka et al., 1999; Vijverberg, 1976). The similarity of phenotypic response to both factors may suggest that there is a universal response pathway to both cyanobacteria presence and low abundance of food. One could speculate that *Daphnia* may perceive the presence of cyanobacteria as being under conditions of food shortage, and the observed changes of phenotype are, in fact, a response to low food quantity, not to the presence of cyanobacteria itself. For instance, in the presence of cyanobacteria, feeding (specifically ingestion) rate is decreased (Rohrlack et al., 2001), and cyanobacteria cells contain protease inhibitors, which may impede the digestion of food (Schwarzenberger et al., 2010). Thus, feeding process is already hampered at the stage of digestion/ingestion of food particles. Moreover, the presence of cyanobacteria can induce changes of *Daphnia* filter screen morphology similar to changes observed in *Daphnia* restricted by low food quantity (e.g., Lampert & Brendelberger, 1996). In the presence of cyanobacteria, enlargement of the filter screen area (Ghadouani & Pinel‐Alloul, 2002) and decrease of the mesh size (Bednarska & Dawidowicz, 2007) was observed. Reducing the mesh size decreases the mechanical interference of filaments in the filtration process (Bednarska & Dawidowicz, 2007), but at the same time, such morphological modifications can be regarded as an adaptation to low food conditions since it facilitates feeding on small food particles such as bacteria, thus allowing *Daphnia* to exploit additional food sources and, at least partially, avoid starvation (see review by Bednarska, 2006).

Still, low food concentration generally imposes different constraints on an animal's fitness than low food quality does. While low food quantity limits the amount of energy which can be allocated into growth and reproduction, the lack of essential compounds in a cyanobacteria‐based diet might critically disturb physiological and metabolic pathways controlling these processes. It has been shown that on a pure cyanobacteria diet, *Daphnia* can grow, but reproduction is hampered, which highlights the importance of biochemical composition of cyanobacteria‐based diet (Martin‐Creuzburg et al., 2005).

Furthermore, *Daphnia* show a variety of adaptive responses, specific under particular environmental regimes (e.g., Gliwicz, 2003; Jiang et al., 2014; Lampert, 2006; Tautz, 2011), thus it is likely that the animals can distinguish between the stress caused by a shortage of food and the stress caused by low‐quality food. Lukas and Wacker (2014a,b) provided evidence of such factor‐specific responses by tracking the changes in carbon (C) metabolism in *Daphnia* exposed to food of different quantity and quality. It was shown that under high quantity of low‐quality food (in terms of cholesterol and EPA), daphnids increased discharge of excess C through increased feces release when compared to individuals fed with low quantity of high‐quality food.

Under food shortage conditions, *Daphnia* produce few but larger‐sized eggs, and therefore larger neonates, than in optimal conditions (Bartosiewicz et al., 2015; Guisande & Gliwicz, 1992; Trubetskova & Lampert, 1995). In contrast, eggs and newborns produced by cyanobacteria‐fed individuals can reach the same size or are smaller than those produced by well‐fed females (Schwarzenberger & Von Elert, 2012; Wacker & Martin‐Creuzburg, 2007). However, the potential differences in per‐offspring investment of daphnids facing conditions of low food quantity and low quality has not yet been addressed within the same study.

The first aim of this study was to test the hypothesis stating that the reproductive strategy (i.e., investment in numbers versus size of the offspring) will be different in *Daphnia* exposed to (i) a low quantity of high‐quality food, and (ii) an unrestricted quantity of low‐quality cyanobacterial food.

The second goal was to test which food level (i.e., how strong dietary restriction) causes similar changes in *Daphnia* life history as does the diet composed of cyanobacteria. Additionally, inter‐clonal differences in the reproductive strategies under created food regimes were analyzed.

## 2 MATERIALS AND METHODS

The experiment was performed with individuals of five clones of *Daphnia magna* Strauss (clones: D1, D2, D3, D4, and B1). Clones originated from the library of clones maintained in the Department of Hydrobiology, University of Warsaw. All clones were originally hatched from ephippia, to represent natural variability within *Daphnia* population (in contrast to clones isolated from water column, which could be preselected by the current conditions of the lake/pond). Clones D1–D4 were isolated in the laboratory from sediments originating from Binnensee (Northern Germany), and clone B1 originated from Oud Heverlee Pond, Belgium (hatched in the Laboratory of Aquatic Ecology, Katholieke Universiteit Leuven). The clones were picked at random from the pool of clones originating form permanent, eutrophic, water bodies, inhabited by fish, in which cyanobacteria presence has been observed. The clones were isolated at least 1 year prior to the study and were maintained at room temperature (19–22°C) in laboratory batch cultures, fed with *Acutodesmus obliquus* (SAG 276‐3a, formerly known as *Scenedesmus obliquus* or *S. acutus f. alternans*). *A. obliquus* is a freshwater green alga with cylindrical cells of ~5 × ~15 μm in size, living either as single cells or forming coenobia of four or eight cells. *A. obliquus* is considered a good quality food due to its high nutritional value and the good handling properties of its cells.

A single female from each clonal culture was selected to establish the clonal lineage. Before the experiment started, clonal lineages were cultured for at least four generations, and only second‐clutch offspring was used for further culture. Animals were cultured under standardized conditions, that is, in a temperature‐controlled water bath (20°C ± 0.5°C), summer photoperiod (16 L:8D), fed daily with *A. obliquus* (1.5 mg C_org_ L^−1^), in density of 1 ind × 50 ml^−1^ of medium, with the culture medium changed every second day.

The experimental media were prepared with aged lake water. Water from the small eutrophic Lake Janówek, located near Warsaw, Poland, was prefiltered through a 1‐μm pore size filter and stored in a large, aerated tank for 2 weeks before use. Immediately before preparing the final experimental media, the water was re‐filtered through a 0.2‐μm ceramic filter and enriched with a suspension of food—either the green alga *A. obliquus* or the filamentous, nontoxic cyanobacterium *Raphidiopsis raciborskii* (strain SAG 1.97, basionym *Cylindrospermopsis raciborskii*). The lack of acute toxicity of this strain of *R. raciborskii* was confirmed in a standard toxicity test with *Daphnia* (Bednarska et al., 2014). Moreover, HPLC analyses reviled the lack of cylindrospermopsin and three homologues of microcystin (MC‐RR, MC‐YR, MC‐LR) in this strain of *R. raciborskii* (SAG 1.97; Wejnerowski et al., 2018). Thus, the effect of toxicity of cyanobacteria was excluded from this study. *R. raciborskii* filaments had a length of 109.4 ± 67.6 μm (mean ± SD), median of 93.5 ± 4.2 μm, with the filament lengths ranging from 29 to 276 μm, wherein on average 43 ± 5% of filaments were shorter than 60 μm. *Daphnia* prefer to consume food particles not exceeding 50–60 μm in size (Bloem & Vijverberg, 1984; Gliwicz, 1980); therefore, it was expected that the filaments used in the study were not only of low nutritive value, but the long filaments in the experimental medium would mechanically interfere with the food gathering process.

Both green algae and cyanobacteria were cultured in semi‐continuous cultures on Z/4 medium (Zehnder & Gorham, 1960), at room temperature (20°C ± 0.5°C), with constant illumination and aeration, with exchange rate of ¼ of the volume daily. The organic carbon content of *R. raciborskii* and *A. obliquus* were estimated by converting photometric light extinction at 800 nm to organic carbon according to empirically established carbon‐extinction regressions.

Daphnids were subjected to five dietary regimes: one cyanobacteria‐based diet treatment (“CY”) and four green algae‐based diet treatments (“GA”) in a gradient of concentration of ca. threefold increase each step, from food level slightly above the threshold concentration for growth for *Daphnia magna* (Gliwicz, 1990a), to a concentration above incipient limiting level, at which the egg production curve reaches a plateau (Lampert, 1978). The dietary regimes were labeled as follows: (i) a “low quality diet” of filaments of nontoxic *R. raciborskii* offered at a concentration of 1.5 mg C_org_ L^−1^ (“1.5 CY”); (ii) a strong dietary restriction of green algae *A. obliquus* offered at a concentration of 0.05 mg C_org_ L^−1^ (“0.05 GA”); (iii) moderate dietary restriction of green algae *A. obliquus* offered at a concentration of 0.15 mg C_org_ L^−1^ (“0.15 GA”); (iv) mild dietary limitation of green algae *A. obliquus* offered at a concentration of 0.5 mg C_org_ L^−1^ (“0.5 GA”); (v) an unrestricted diet of green algae *A. obliquus* offered at a concentration of 1.5 mg C_org_ L^−1^ (“1.5 GA”). The concentration of 1.5 C_org_ L^−1^ was deliberately chosen as a concentration of quantitatively unrestricted diet. Lampert (1978) had shown that incipient limiting level (ILL) for egg production of food particles <50 μm is ~0.7 mg C_org_ L^−1^ and the concentration of 1.5 mg C_org_ L^−1^ used in the study was more than 2 times higher than ILL. Thus, in the provided biomass of *R. raciborskii* the amount of edible, short filaments (43 ± 5% of all filaments) was close to ILL (to avoid effect caused by quantity restriction). The presence of long filaments in the medium allows to combine the effect of interference of food gathering process with low nutritional value of cyanobacteria.

*Daphnia* neonates (within 12 h of birth) of the second clutch of each of the five tested clones served as experimental individuals. Experimental females were separated from their mothers and randomly assigned to one of the experimental treatments; there were 10 replicates in each treatment (dietary regime). Additionally, 30 newborns of each of the five clones were dried and weighed to determine their initial (day zero) body mass. Females were kept separately in 100‐ml glass vessels in one of the five experimental media. Media were changed daily. The experiment was performed under 20°C (±0.5°C), and 16 L:8D light conditions. Age and size at first reproduction, growth rate, the number of eggs, individual egg size (expressed as egg volume), and the clutch size (expressed as clutch volume) were measured.

The experiment ended when eggs of the first clutch were deposited in the brood chambers. Then, for each female, the number of eggs was counted intravitally (from the 3D image under the binocular microscope). Immediately after the eggs were counted, females were photographed (binocular microscope equipped with digital camera), dried (24 h, 60°C), and weighed to calculate growth rate (Orion Cahn C‐35 Ultra‐Microbalance, Thermo Electron Corporation, USA). Dry masses were converted into growth rates per day using the formula *g*_*i*_ = (ln[*M*_*t*_] − ln[*M*_0_])/*t*; (Lampert & Trubetskova, 1996), where *M*_0_ and *M*_*t*_ are the dry masses of the animals at day zero and at the end of the experiment, respectively, and *t* is the duration of the experiment (age at first reproduction, in days). Body length of *Daphnia* and the two dimensions of the egg were measured from photographs using NIS‐Elements software (Nikon). For each female, up to five eggs were measured, though females with restricted food quantity and quality often had fewer than five eggs in their brood chambers. Mothers were inspected daily for the presence of eggs in the brood chamber, thus the developmental stages of eggs may have differed by up to 24 h. To account for this, the egg development stage was assessed according to parameters proposed by Gulbrandsen and Johnsen (1990). The 24‐h interval under thermal conditions of the experiment limited the variability to 1st–2nd developmental stage. Only eggs in the 2nd development stage were measured, that is, eggs still enveloped in the first membrane, with prominent granulation of egg mass. Two dimensions of an egg were used to calculate the egg volume according to formula for the volume of a prolate ellipsoid: *V* = 4/3 π *a b* ^2^, where “*a*” is the longer “radius,” and “*b*” is shorter “radius” of the egg. Clutch volume was calculated by multiplying the mean volume of egg by the number of eggs produced by a given female.

The effects of food regime and clonal affiliation on life history parameters were analyzed using two‐way ANOVA, followed by posthoc Tukey HSD tests; all analyses were performed with Statistix 9.0 software.

## 3 RESULTS

**FIGURE 1:**
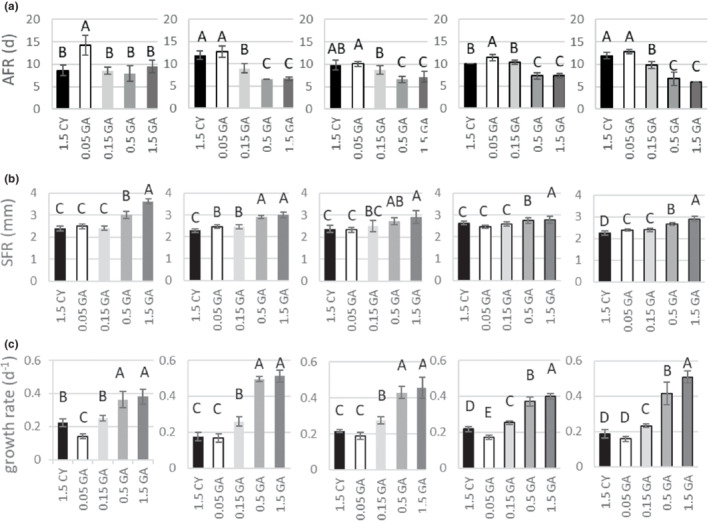
Growth‐related traits. Age at first reproduction (AFR), size at first reproduction (SFR), and individual growth rate (g_*i*_), (mean ± SD) of individuals of five clones (D1, D2, D3, D4, and B1) of *D. magna* cultured under five food regimes: Numbers indicate the food concentration expressed in mg C L^−1^, CY‐cyanobacterium, GA, green algae. Letters above the bars indicate the homogeneous groups based on Tukey HSD test performed for each clone separately.

**FIGURE 2:**
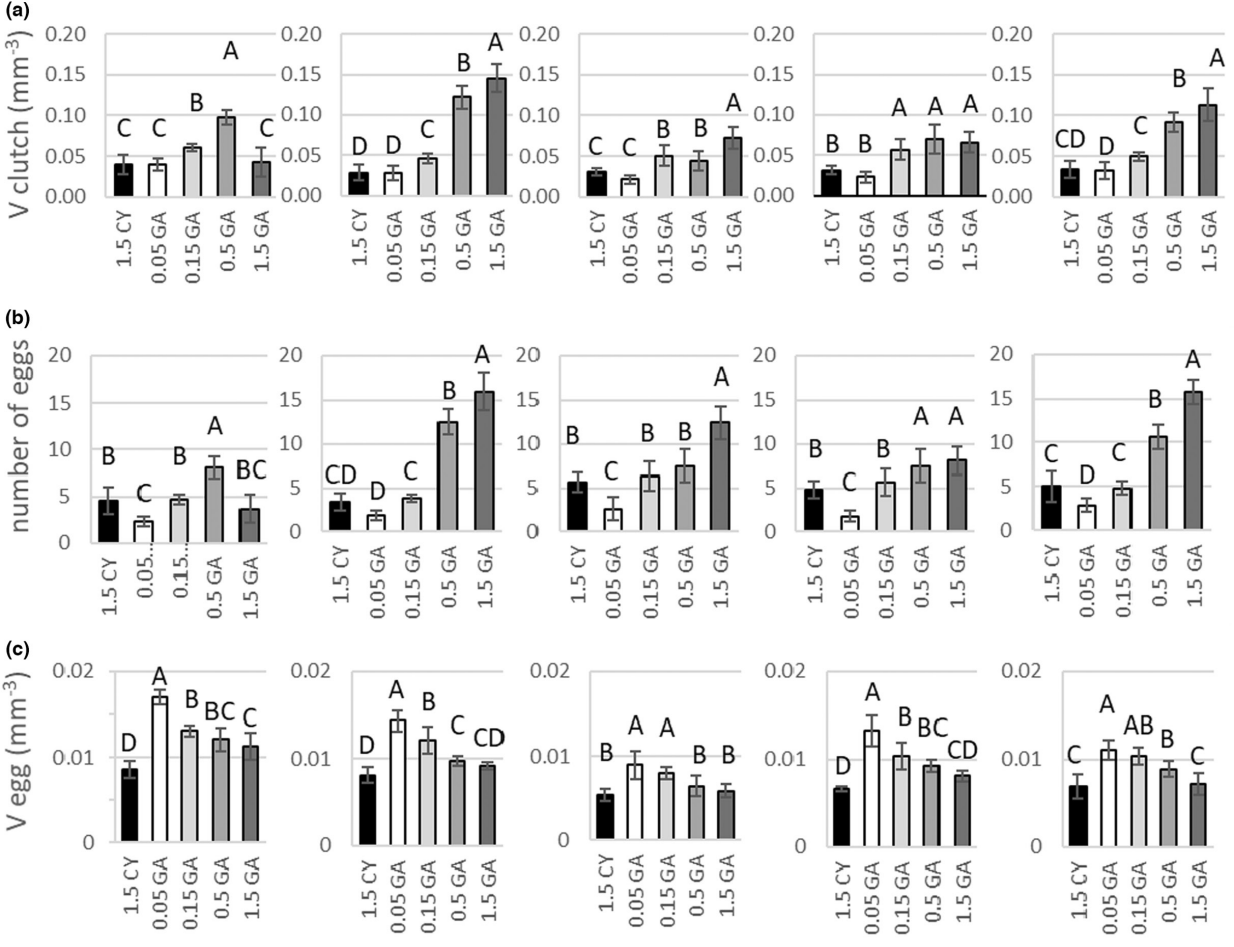
Reproduction‐related traits. Clutch volume (V clutch), number of eggs (eggs #), and egg volume (V egg) (mean ± SD) of individuals of five clones (D1, D2, D3, D4, and B1) of *D. magna* cultured under five food regimes: numbers indicate the food concentration expressed in mg C_org_ L^−1^, CY‐cyanobacterium, GA‐ green algae. Letters indicate the homogeneous groups based on Tukey HSD test performed for each clone separately.

Food type, clonal affiliation, and the interaction of these two factors were significant sources of variation of all observed life history parameters (Two‐way ANOVA; Figures 1 and 2; Table 1). Animals on unrestricted diet (“1.5 GA”; 1.5 mg C L^−1^ of green algae *A. obliquus*) generally matured fastest (on ~7.4 day), reached largest body size (~3.05 mm), had the highest growth rate (~0.45 day^−1^), and produced the highest number (~11.2 eggs per female) of second smallest or smallest sized eggs (~0.008 mm^−3^), which resulted in the greatest clutch volume (~0.09 mm^−3^; Tukey HSD; α = 0.01; Figures 1 and 2). Although in most cases the animals on unrestricted diet produced clutches of the largest volume, there were interclonal differences in absolute per‐clutch investment (mean clutch volume in order from smallest to the largest: D1 = 0.042 mm^−3^, D4 = 0.066 mm^−3^, D3 = 0.072 mm^−3^, B1 = 0.113 mm^−3^, D2 = 0.145 mm^−3^; Figure 2a). In general, size at reproduction, growth rate, clutch volume, and number of eggs increased, and age at reproduction and egg size decreased along with increasing quantity of food (Tukey HSD; α = 0.01; Figure 1a–d). Within a clone, the key life history parameters, that is, growth rate and per‐clutch investment (clutch volume) of individuals cultivated under cyanobacterial diet (1.5 CY; 1.5 mg C_org_ L^−1^; mean growth rate: D1 = 0.223 day^−1^, D2 = 0.176 day^−1^, D3 = 0.217 day^−1^, D4 = 0.217 day^−1^, B1 = 0.186 day^−1^, mean clutch volume: D1 = 0.039 mm^−3^, D2 = 0.028 mm^−3^, D3 = 0.030 mm^−3^, D4 = 0.031 mm^−3^, B1 = 0.034 mm^−3^) fit between the mean values of those life history parameters of animals kept in the two lowest food concentrations (i.e., 0.05 mg C_org_·L^−1^ and 0.15 mg C_org_·L^−1^), but were more alike and almost always not significantly different from the values of those parameters for the females receiving the lowest food concentration tested (0.05 mg C_org_·L^−1^; Tukey HSD; α = 0.01; Figures 1c and 2a; mean growth rate: D1 = 0.142 day^−1^, D2 = 0.168 day^−1^, D3 = 0.189 day^−1^, D4 = 0.170 day^−1^, B1 = 0.157 day^−1^, mean clutch volume: D1 = 0.040 mm^−3^, D2 = 0.027 mm^−3^, D3 = 0.021 mm^−3^, D4 = 0.023 mm^−3^, B1 = 0.032 mm^−3^). The number of eggs produced by mothers in “1.5 CY” treatment (mean: 4.7 eggs per female) was not significantly different from the number of eggs produced by mothers in “0.15 GA” (mean: 5.0 eggs per female). However, the egg size (mean: for 1.5 CY treatment ~0.007 mm^−3^) was not only significantly smaller than that of eggs produced by mothers of both “0.05 GA” and “0.15 GA” treatments (0.013 and 0.011 mm^−3^, respectively), but the same size or smaller than eggs produced even by mothers on unrestricted diet (Figure 2b,c).

**TABLE 1:** Results of analysis of variance (two‐way ANOVA) testing the effects of clone and food type on key life history parameters of *Daphnia*: Age at first reproduction (AFR), size at first reproduction (SFR), growth rate (*g*_*i*_), clutch volume (*V* clutch), number of eggs (# eggs), and egg volume (*V* egg).

Life history parameter	Source of variance	*df*	*F*	*p*
Age (AFR)	Food (F)	4	232.47	<.00001
	Clone (C)	4	8.97	<.00001
	F × C	16	14.60	<.00001
	Error	212		
Size (SFR)	Food (F)	4	220.01	<.00001
	Clone (C)	4	24.28	<.00001
	F × C	16	15.07	<.00001
	Error	211		
g_i_	Food (F)	4	875.48	<.00001
	Clone (C)	4	23.93	<.00001
	F × C	16	15.29	<.00001
	Error	203		
*V* clutch	Food (F)	4	261.65	<.00001
	Clone (C)	4	49.16	<.00001
	F × C	16	33.24	<.00001
	Error	209		
# eggs	Food (F)	4	336.34	<.00001
	Clone (C)	4	44.79	<.00001
	F × C	16	33.98	<.00001
	Error	212		
*V* egg	Food (F)	4	198.65	<.00001
	Clone (C)	4	161.16	<.00001
	F × C	16	4.48	<.00001
	Error	209		

Individuals of the D1 clone performed significantly different from individuals of other clones (Tukey HSD; α = 0.01, Figures 1 and 2). Individuals of D2 clone had the highest mean growth rate and clutch volume. The greatest interclonal differences were observed in mean egg size (Figure 2c).

## 4 DISCUSSION

### 4.1 Differences in reproductive strategy between daphnia restricted by food quantity and quality

The biggest differences between food treatments could be seen in reproduction‐related traits. The per‐clutch investment observed in cyanobacteria‐fed and underfed animals was not significantly different (Figure 2), but the strategy of resource allocation into respective components of this investment (i.e., number vs. size of eggs) differed substantially (Figure 2b,c). Females experiencing restricted food quantity increased their per‐offspring investment, that is, produced fewer but larger eggs than mothers that were fed unlimited amounts of low‐quality food. Animals fed with cyanobacteria produced more eggs than mothers limited by strong dietary restriction (0.05 mg C_org_ L^−1^), and the number of eggs was in general not significantly different from the number of eggs produced by the mothers experiencing moderately restricted diet (0.15 mg C_org_ L^−1^), but the egg size was the same as in mothers on unrestricted diet. In accordance with *r*/K selection theory, it can be stated the food quantity‐restricted females apply a strategy leaning toward K, while cyanobacteria‐fed daphnids adopt *r* strategy (they do not increase provisioning of the eggs with storage materials). It can be argued that both strategies of resource allocation are adaptive, as food‐limited mothers have been shown to produce larger eggs (Bartosiewicz et al., 2015; Guisande & Gliwicz, 1992; this study). Offspring hatched from larger eggs have certain advantages. First, the neonates born from larger eggs are equipped with a greater amount of storage materials (Guisande & Gliwicz, 1992), and thus have a greater capability to withstand starvation (Gliwicz & Guisande, 1992). Second, as the size at birth correlates with the size of the eggs the neonates are hatched from (Guisande & Gliwicz, 1992), and because filtration efficiency is positively correlated with the size of *Daphnia* (Gliwicz, 1990b), the relatively large size at birth allows for higher filtration efficiency. Thus, under condition of low food quantity, larger‐sized neonates can gain an advantage over smaller individuals.

This study demonstrates that instead of increased “per‐offspring” investment, cyanobacteria‐fed mothers invested more in offspring number (*r* strategy), dividing the available resources into a greater number of relatively small eggs. Wacker and Martin‐Creuzburg (2007) hypothesized that cyanobacteria‐fed mothers would modify the size and biochemical composition of the eggs in a similar way as underfed mothers do. They postulated that increased allocation of storage materials—PUFA and sterols—is adaptive, since it should improve the survival chances of the neonates when the cyanobacterial diet does not provide a sufficient amount of these compounds. However, my results are consistent with those obtained by Wacker and Martin‐Creuzburg (2007) and Sperfeld and Wacker (2009) and do not support their initial hypothesis. Although the amount of PUFAs was higher in eggs than in maternal somatic tissue, the amount of almost all measured PUFAs allocated into eggs was not relatively higher but proportional to the availability of those compounds in the mothers' diet (Sperfeld & Wacker, 2009; Wacker & Martin‐Creuzburg, 2007). This could indicate a lack of increased egg provisioning. However, those authors also showed that the amount of α‐linolenic acid (ALA) and cholesterol allocated into eggs is kept at a constant level even at the expense of decreased content of those compounds in somatic tissue. Increased allocation of only particular compounds highlights their importance for *Daphnia* growth and reproduction and may indicate that although there is no overall increased allocation into eggs by cyanobacteria‐fed individuals, the egg provisioning is limited to very specific, essential compounds.

Moreover, production of larger eggs, which hatch into bigger neonates, does not necessarily translate into future fitness advantages when cyanobacteria are abundant in the environment. It has been suggested that mechanical interference in the filtration process caused by filamentous cyanobacteria is positively body size‐dependent (Gliwicz, 1990a; Gliwicz & Lampert, 1990). The size at birth determines the further growth of an individual including size at first reproduction (Ebert, 1991). Usually, the fecundity of *Daphnia* is positively correlated with body size, and such dependence has been shown for individuals fed both with high and low quantities of green algae (e.g., Bartosiewicz et al., 2015), and for individuals fed with short, relatively easy to handle filaments of cyanobacteria (Bednarska et al., 2014). However, in the presence of long filaments of cyanobacteria, the highest fecundity at first reproduction was achieved not by the largest mothers, but those of intermediate size (Bednarska et al., 2014). Smaller size at birth and subsequently smaller size at reproduction may be regarded as adaptive, since it reduces filtration disturbance caused by the presence of long filaments. However, to support this reasoning, the multigenerational study should be performed.

The production of smaller eggs can potentially be adaptive for another reason, that is, due to the total metabolic demands for embryogenesis of a single individual. In general, the metabolic rate per mass unit increases with decreasing body size (Kleiber, 1947); therefore, smaller embryos have a relatively higher metabolic rate than larger ones. Increased share of respiration in pathways of carbon metabolism was demonstrated in *Daphnia* cultured at high quantity of low‐quality food (low cholesterol; Lukas & Wacker, 2014a). It can be speculated that higher metabolic rate resulting from small egg/embryo size can be a way to get rid of excess carbon under sterol limitation. However, if metabolic costs are expressed not per mass unit, but per individual, the total metabolic demand for embryonic development increases proportionally with egg size (J. Macháček & J. Seďa, personal communication). This is caused by (1) the lower egg mass and (2) shorter development time of smaller eggs (J. Macháček & J. Seďa, personal communication). The positive correlation between the duration of embryonic development and egg/neonate mass has been reported for cladocerans in inter‐specific comparisons (Gillooly & Dodson, 2000; Vijverberg, 1980) and in a clone of *D. galeata* (J. Macháček & J. Seďa, personal communication). However, this explanation requires further investigation, since we know little about the metabolic demands of embryos produced by food quality‐restricted mothers, and how poor provisioning of the egg might affect the embryonic development time.

Direct provisioning of the eggs with storage materials and other essential compounds is not the only way in which mothers can shape the phenotype of their offspring. There are several mechanisms by which *Daphnia* can prepare their offspring for anticipated food conditions via maternal effect. Crowding cues, which forecast the deterioration of feeding conditions, can stimulate *Daphnia* to produce offspring, which are more resistant to starvation (Mikulski, 2006). Mothers can also affect the offspring feeding rate, as it was shown that offspring of food‐restricted mothers can have larger filter screens (Garbutt & Little, 2014). Bigger filter screens can potentially increase the filtering efficiency, thus allowing for more efficient growth. An increased tolerance against toxic cyanobacteria in offspring born to mothers exposed to cyanobacteria has also been demonstrated (Gustafsson et al., 2005; Jiang et al., 2013; Lyu et al., 2015; Radersma et al., 2018). Moreover, there is maternal transfer of increased expression of protease‐encoding genes, and since cyanobacterial cells contain protease inhibitors, this change in gene expression enhances offspring growth and is most certainly adaptive (Schwarzenberger & Von Elert, 2012).

The results also demonstrate that the determination of reproductive effort based solely on the number of eggs can underestimate the real costs borne by females. The number of eggs produced by cyanobacteria‐fed mothers was usually higher than those produced by their conspecifics experiencing strong dietary restriction, but the total investment into a single reproductive event was the same. Future studies should therefore consider both the number and the size of eggs produced, as well as the offspring fitness. Additionally, it was shown that the presence of cyanobacteria in the environment may increase embryo mortality or even cause egg abortion (Bednarska & Slusarczyk, 2013, Wejnerowski et al., 2020); thus, to correctly assess the cyanobacteria‐driven fitness costs, besides the number/size of eggs, the number and size of vital neonates should be taken into consideration as well.

### 4.2 Importance of food quantity vs. quality and quantifying the impact of stress caused by cyanobacteria

In this study, the strength of negative impact of poor‐quality cyanobacterial food on *Daphnia* fitness could be quantified by contrasting the changes of life history of cyanobacteria‐fed *Daphnia* with the life histories of *Daphnia* fed with different amounts of high‐quality food. The results indicate that the presence of cyanobacteria limits the fitness of *Daphnia* to a similar extent as the low (near‐threshold) food concentration does. The mean values of key life history parameters of animals fed with cyanobacteria offered in concentration of 1.5 mg C_org_ L^−1^ were, in general, not significantly different from the analogical parameters of *Daphnia* fed with green algae in 0.05 mg C_org_ L^−1^ concentration.

The lowest concentration of *A. obliquus* used in this study (0.05 mg C_org_ L^−1^) was higher than the threshold food concentration for the growth of *Daphnia magna*, estimated as approximately 0.02 mg C_org_ L^−1^ (Gliwicz, 1990a), to enable successful growth and reproduction of animals, but it is nevertheless a strong dietary restriction (Gliwicz, 1990a). The similarities in life histories of *Daphnia* fed with green algae of this concentration and cyanobacteria‐fed individuals indicates that cyanobacteria presence is a strong selective factor. These results are in agreement with previous findings related to the effect of low food quantity and quality (cyanobacteria presence) on *Daphnia* fitness (Guo & Xie, 2011; Repka et al., 1999; Vijverberg, 1976).

It seems that low food quality and quantity affect the growth‐related life history traits in a similar way. Under conditions of low food quantity and low food quality, individuals of the majority of *Daphnia* clones were of not significantly different size and age at adulthood, and had similar growth rates (Figure 1a–c). A similar pattern was observed for *Daphnia galeata* (Repka et al., 1999) where the juvenile growth rate of individuals fed with a low concentration of high‐quality green algae was the same as the growth rate of daphnids fed with a high concentration of a filamentous cyanobacterium. Also, Lukas and Wacker (2014a) found no significant differences between somatic growth rate of *Daphnia* cultured in low quantity of high‐quality food and high quantity of low‐quality food. This indicates that quantitative and qualitative food limitation is of the same importance.

The most striking evidence of the strength of stress caused by a cyanobacteria diet is not significantly different *per‐clutch* investment in cyanobacteria‐fed animals and in animals cultured at near‐threshold food level (Figure 2a). Individuals on lowest quantity and low‐quality diet were able to allocate the same amounts of resources into reproduction. This indicates that the net amount of resources gathered during the juvenile period of life by individuals limited by low food quantity is approximately the same as by individuals experiencing a cyanobacterial diet.

Although the fitness decrease due to cyanobacteria‐based diet shown in this study is considerably high, the strength of such negative impact on *Daphnia* fitness may still be underestimated. The influence of cyanobacteria depends greatly on both the species/clone of *Daphnia* (e.g., Bednarska et al., 2014; Jiang et al., 2013) and the species/strain of cyanobacteria (Hochmuth & De Schamphelaere, 2014; Wejnerowski et al., 2015), or the combination of these two variables (Lemaire et al., 2012). The vulnerability of *Daphnia* to the presence of cyanobacteria may depend also on other environmental factors, such as temperature (Bednarska et al., 2011; Wejnerowski et al., 2020). Thus, although the detrimental effect of cyanobacteria on *Daphnia* fitness should always be expected, the degree to which they are impacted is highly context‐dependent.

Although interclonal differences have been found in this study, they are expressed rather in responses to food quantity than quality. Individuals of studied clones when cultured on cyanobacteria diet did not differed in per clutch investment (Figure 2a). The important interclonal differences can be found, however, in the age at first reproduction (Figure 1a). Individuals of D1 clone in the cyanobacteria presence matured significantly earlier (on ~8.6 day), when compared to individuals of clone D3 and D4 which matured later (~10 days) or clones D2 and B1which reproduced at the latest (~12 days). This can significantly impact the growth rate (*r*) of the clone and determine the genotype frequency in the population. The significant interclonal differences in response to food quantity found here support findings of earlier study conducted with three out of five clones used in this study (Pietrzak et al., 2010). The age‐related changes in reproductive value and life history trade‐off (life span vs. early reproduction effort) revealed the highest reproductive value at food level of 0.5 mg C_org_·L^−1^ for individuals of clone D1, at 1.5 mg C_org_·L^−1^ for B1, and at 4.5 mg C_org_·L^−1^ for D2. This suggests the existence of “specialists,” which perform best at different food levels. Rapidly changing food conditions may, thus, grant the competitive advantage to individuals of different clones at any given time leading to seasonal sequence of dominance within *Daphnia* population.

It has been shown that while the inadequate stoichiometry of green algae reduced the quality of food, an increase in quantity of P‐limited algae did not improve the growth and reproduction of daphnids (Sarpe et al., 2014). Thus, it can be concluded that the quantity of food cannot fully compensate for its low quality. However, when the presence of cyanobacteria is responsible for the reduction in food quality, an increase of cyanobacteria concentration may lead to even more pronounced fitness losses. The negative effect of cyanobacteria is positively concentration‐dependent, so increasing the concentration of cyanobacterial filaments in the environment will likely lead to more severe effects on *Daphnia* fitness (Zhou et al., 2020). Further evidence is provided by the observation that the density of daphnids decreases with increasing concentration of cyanobacteria (e.g., Ghadouani et al., 2003; Threlkeld, 1979; Webster & Peters, 1978).

## AUTHOR CONTRIBUTIONS

**Anna Bednarska:** Conceptualization (lead); data curation (lead); formal analysis (lead); funding acquisition (lead); investigation (lead); methodology (equal); project administration (lead); resources (lead); supervision (lead); validation (lead); visualization (lead); writing – original draft (lead); writing – review and editing (lead).

## CONFLICT OF INTEREST

The author declare that they have no conflict of interest.

### OPEN RESEARCH BADGES

This article has earned an Open Data badge for making publicly available the digitally‐shareable data necessary to reproduce the reported results. The data is available at https://doi.org/10.18150/PVTRYI.

## Data Availability

The data that support the findings of this study are openly available in RepOD at https://doi.org/10.18150/PVTRYI.
